# Implications of STAT3 and STAT5 signaling on gene regulation and chromatin remodeling in hematopoietic cancer

**DOI:** 10.1038/s41375-018-0117-x

**Published:** 2018-03-27

**Authors:** Bettina Wingelhofer, Heidi A. Neubauer, Peter Valent, Xiaonan Han, Stefan N. Constantinescu, Patrick T. Gunning, Mathias Müller, Richard Moriggl

**Affiliations:** 1 Institute of Animal Breeding and Genetics, University of Veterinary Medicine Vienna, Vienna, Austria; 2 Ludwig Boltzmann Institute for Cancer Research, Vienna, Austria; 3 Department of Internal Medicine I, Division of Hematology and Hemostaseology, Ludwig Boltzmann-Cluster Oncology, Medical University of Vienna, Vienna, Austria; 4 Division of Gastroenterology, Hepatology and Nutrition, Cincinnati Children’s Hospital Medical Center (CCHMC), Cincinnati, OH USA; 5 Key Laboratory of Human Disease Comparative Medicine, the Ministry of Health, Institute of Laboratory Animal Sciences (ILAS), Chinese Academy of Medical Science (CAMS) and Peking Union Medical College (PUMC), Beijing, China; 6 Ludwig Institute for Cancer Research Brussels, Brussels, Belgium; 7 Université Catholique de Louvain and de Duve Institute, Brussels, Belgium; 8 Department of Chemical & Physical Sciences, University of Toronto Mississauga, Mississauga, ON Canada; 9 Department of Chemistry, University of Toronto, Toronto, ON Canada; 10 Medical University Vienna, Vienna, Austria

## Abstract

STAT3 and STAT5 proteins are oncogenic downstream mediators of the JAK–STAT pathway. Deregulated STAT3 and STAT5 signaling promotes cancer cell proliferation and survival in conjunction with other core cancer pathways. Nuclear phosphorylated STAT3 and STAT5 regulate cell-type-specific transcription profiles via binding to promoter elements and exert more complex functions involving interaction with various transcriptional coactivators or corepressors and chromatin remodeling proteins. The JAK–STAT pathway can rapidly reshape the chromatin landscape upon cytokine, hormone, or growth factor stimulation and unphosphorylated STAT proteins also appear to be functional with respect to regulating chromatin accessibility. Notably, cancer genome landscape studies have implicated mutations in various epigenetic modifiers as well as the JAK–STAT pathway as underlying causes of many cancers, particularly acute leukemia and lymphomas. However, it is incompletely understood how mutations within these pathways can interact and synergize to promote cancer. We summarize the current knowledge of oncogenic STAT3 and STAT5 functions downstream of cytokine signaling and provide details on prerequisites for DNA binding and gene transcription. We also discuss key interactions of STAT3 and STAT5 with chromatin remodeling factors such as DNA methyltransferases, histone modifiers, cofactors, corepressors, and other transcription factors.

## Introduction

**Fig. 1:**
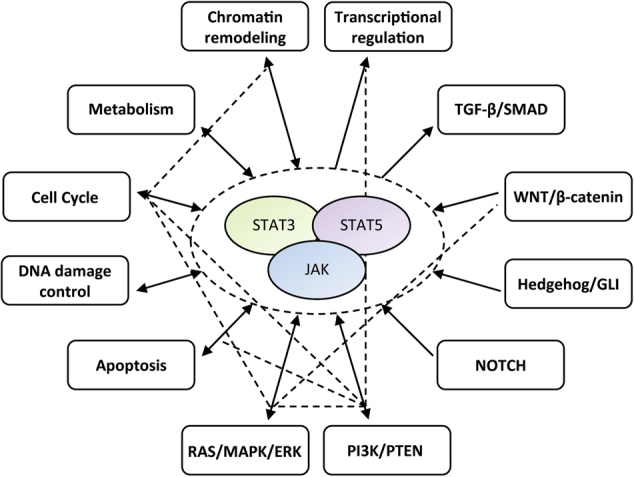
JAK–STAT signaling is interconnected with core cancer pathways. Core cancer pathways modulate or converge on the JAK–STAT pathway to control cell survival, differentiation, proliferation, and metabolism in response to extracellular stimuli. Arrows indicate either uni- or bidirectional interconnections of core cancer pathways. Dotted lines represent interactions independent of JAK–STAT signaling

Over the past decade, extensive next-generation sequencing (NGS) efforts and comparative data integration have provided insights into the mutational landscape of human cancer genome coding exons. These studies defined ~140 different cancer driver genes within 12 core cancer pathways that, when mutated, can promote tumorigenesis [1]. These cancer driver genes regulate three main cellular processes: cell fate, cell survival, and genome maintenance [1]. Defining these core cancer pathways and acknowledging their multifaceted and interconnected nature has helped to stratify the complex genetics of cancer, which has significantly influenced both the intellectual approach of cancer biologists and the pharmaceutical development of specific inhibitors. However, recent research focuses not only on mutations that modify the specific key players of the core cancer pathways, but also on mutations of chromatin regulatory sites within non-coding regions of DNA. Mutations in these regions very often result in epigenetic changes that influence gene expression in cancer cells. The interplay between mutations in the core cancer pathways and changed chromatin composition and its influence on transcription is considered as one of the most relevant concepts in current basic cancer research. Many of the genes that define core cancer pathways are directly involved in or converge in the Janus kinase/signal transducer and activator of transcription (JAK–STAT) pathway (Fig. 1). Extensive studies utilizing STAT knockout mice have revealed the mechanisms of canonical JAK–STAT signaling that are influenced by several regulatory layers [2]. These include cell-type-specific expression and cellular effector abundancies, differential affinity to receptors and their cognate tyrosine kinases (TKs), activity regulation by different post-translational modifications, nucleoplasmic shuttling, recycling by phosphatases, and the interactions with different co-regulators. Functional differences in the STAT proteins might be attributed to their ability to only recognize regulatory sequences in certain contexts, such as composite promoter elements or upon specific chromatin configurations. In fact, evidence is emerging that specific chromatin remodeling is required for STAT binding to a subset of loci [3]. It is suggested that other oncogenic transcription factors, such as steroid receptors, which bind to multiple sites nearby genes without having a transcriptional function, may play a role in the reconstruction of the genome organization [4]. Similarly, STATs may be directly involved in the regulation of chromatin topology [5], not only directly by binding to canonical-binding sites in the active, tyrosine phosphorylated form, but also through their ability to form oligomers and to exert functions in the cytoplasm and nucleus as unphosphorylated dimers (uSTAT). However, the molecular mechanisms governing transcription factor-mediated structural rearrangements in the genome are still poorly understood.

Recent technological advances in molecular biology, resulting from the rise in “next-generation” techniques, have revealed new aspects of JAK–STAT signaling including recurrent somatic mutations of STATs, a plethora of novel DNA-binding sites, post-translational modifications (PTMs), and protein–protein interactions (PPIs), all of which have significant impact on the chromatin landscape. These findings have led to new mechanistic insights into the molecular processes of tumorigenesis that are not only induced by constitutively active STAT, but also regulated by non-canonical STAT functions. STAT3 and STAT5 are of particular interest because they are not only activated by a wide variety of ligands that control proliferation, survival, cell–cell communication, adhesion, and angiogenesis [6], but their dysregulation also facilitate tumor progression in various human cancers, particularly leukemia and lymphomas [7, 8] (Table 1). STAT5 refers to two highly related genes, STAT5A and STAT5B, which are both found on human chromosome 17 [9]. The STAT3 gene lies adjacent to the STAT5 locus and generates two isoforms STAT3α and STAT3β by alternative splicing. Although we acknowledge the different biochemical and biological properties of the STAT3 and STAT5 isoforms [2, 10], this is not in the focus of the following review. In most cells, STAT3 and STAT5 are activated by different mechanisms and bind to distinct loci to regulate specific target gene expression [11, 12]. However, STAT3 and STAT5 proteins can also bind to the same regulatory oncogenic loci resulting in compensatory or antagonistic signaling [13, 14]. Functional redundancy is particularly evident in definitive erythropoiesis, where STAT3 compensates for a loss of STAT5 [14]. Furthermore, competitive binding of STAT3 and STAT5 is best exemplified by the regulation of BCL6 expression, as discussed further below [13]. Additionally, STAT3 and STAT5 were both shown to contain gain-of-function mutations in hotspot residues in their SH2 domain or their extreme C terminus [15]. As such, they were defined as driver genes predominantly in peripheral T cell leukemia/lymphoma (PTCL) or T cell prolymphocytic leukemia/lymphoma (T-PLL), rare but aggressive forms of T cell neoplasia. Interestingly, STAT3 and STAT5 mutations in hematopoietic cancers exist in a mutually exclusive manner and often co-occur with mutations in DNA-modifying enzymes such as DNA methyltransferase 1/3A (DNMT1/3A), Ten-eleven translocation methylcytosine dioxygenase 1/2 (TET1/2), or isocitrate dehydrogenase 2 (IDH2), and corepressor molecules with histone deacetylase (HDAC) activity such as BCL6 Corepressor (BCoR)/Nuclear receptor corepressor 1/2 (NCoR1/2) [16].

**Table 1:** STAT3 and STAT5 activation in hematopoietic diseases

Disease	Cell type	STAT activity
*Leukemia*		
ALL	B or T lymphocytes	STAT5, STAT3
CLL	B lymphocytes	STAT3
Multiple myeloma	Plasma cells	STAT3
AML	Myeloid cells	STAT5, STAT3
APL	Promyelocytes	STAT5
Erythroleukemia	Erythroleukemia/blast cells	STAT5
AMKL	Megakaryocytes	STAT5
EMS/SCLL	Myeloid progenitor cells	STAT5
CML	Granulocytes	STAT5
PV	Erythrocytes	STAT5
ET	Megakaryocytes	STAT5
Idiopathic myelofibrosis	Megakaryocytes	STAT5
SCN	Promyelocyte/myelocyte	STAT5
CMML	Monocytes	STAT5
Mastocytosis	Mast cells/basophils	STAT5
CEL	Eosinophils	STAT5
*Lymphoma*		
B cell lymphoma	B cells	STAT5, STAT
EBV-related and Burkitts lymphoma	B cells	STAT3
Hodgkin lymphoma	T/B cells	STAT5, STAT3
CTCL	T cells	STAT5, STAT3
ALCL	T cells	STAT5, STAT3
LGL leukemia	T/NK cells	STAT3
HTLV-1 infection	T cells	STAT5, STAT3
HVS infection	T cells	STAT3

*ALL* acute lymphocytic leukemia, *CLL* chronic lymphocytic leukemia, *AML* acute myeloid leukemia, *AMl* acute promyelocytic leukemia, *AMKL* acute megakaryoblastic leukemia, *EMS/SCLL* 8p12 myeloproliferative syndrome/stem cell leukemia-lymphoma syndrome, *CML* chronic myeloid leukemia, *PV* polycythemia vera, *ET* essential thrombocythemia, *SCN* severe congenital neutropenia, *CMML* chronic myelo-monocytic leukemia, *CEL* chronic eosinophilic leukemia, *EBV* Epstein–Barr virus, *CTCL* cutaneous T cell lymphoma, *ALCL* anaplastic large cell lymphoma, *LGL* large granular lymphocyte, *HTLV* human T cell lymphoma virus, *HVS* herpesvirus saimiri

Given that chromatin remodeling and the JAK–STAT pathway are both core cancer pathways (Fig. 1) and are often co-mutated in various human cancers, it is of great interest to understand how these signaling nodes are interconnected. In the following, we will review different functions of STAT3 and STAT5 in controlling gene regulation and genome integrity during health or disease, specifically in the context of known protein–protein interaction partners, particularly those involved in chromatin remodeling.

## Cell-type-specific STAT5 target gene regulation

STAT proteins act as transcriptional activators upon phosphorylation of a conserved tyrosine residue at the C terminus followed by translocation into the nucleus, where they bind to DNA and activate target gene transcription [2]. STAT-binding sites are usually found in enhancer and promoter regions as well as first introns of target genes and characterized by clusters of conserved motifs with an interferon gamma-activated site (GAS)-like core sequence (TTCT/CNA/GGAA). The murine or human genomes comprise ~1 million GAS-like sequences, where ~10% are indeed bound by STAT molecules. Close proximity of multiple binding sites leads to binding of additional STAT molecules resulting in increased transcriptional activity [17]. STAT5 expression is often upregulated in cancer, and this increased activity can promote additional STAT binding to less conserved GAS consensus elements. For example, growth hormone (GH)-induced STAT5 DNA binding was observed at 13,278 sites containing GAS motifs within the genome of mouse embryonic fibroblasts (MEFs), but enhanced STAT5 expression lead to a significant increase in genome-binding sites, where up to 72,000 sites were mapped upon 20-fold overexpression of STAT5A [18]. Of the STAT5-binding peaks, 50% coincided with GAS motifs, confirming that STAT5 binds to these specific sequence motifs. However, the nature of STAT5 binding to sequences without a bona fide GAS motif is not clear.

Overall, STAT5A dimers do not bind as efficiently to DNA as STAT5B dimers, which can also recognize 4 bp spaced motifs of TTCT/CN2A/GGAA. STAT5A preferentially forms tetramers even when two weak STAT5 affinity sites are in close proximity. Tetramerization has not been prominently reported for STAT5B, however upon heterodimerization with STAT5A, STAT5B can efficiently take part in the formation of DNA oligomers that are bound at enhancer or promoter regions. There are several amino acid differences in both the oligomerization and DNA-binding domains of STAT5A/B and these could impact DNA-binding efficiency as dimers or oligomers. However, to date there are no crystal structure analyses to provide a deeper understanding of STAT5A or STAT5B oligomer configuration.

**Fig. 2:**
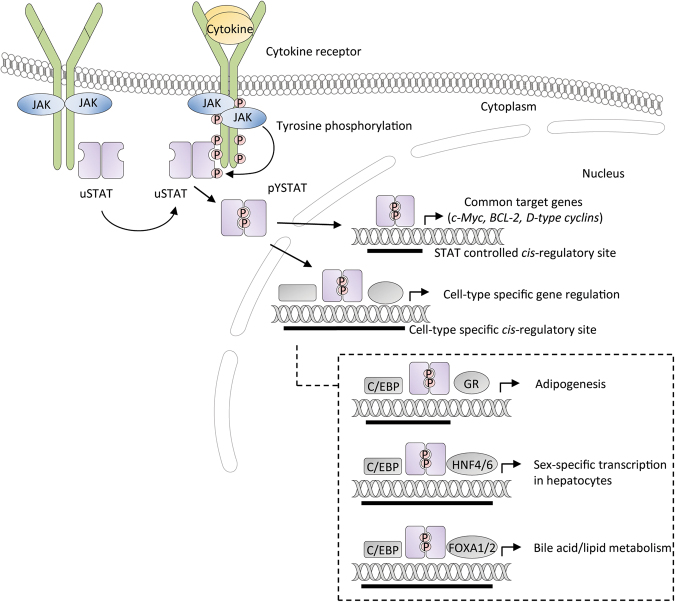
STAT5 mediates common and cell-type-specific gene regulation. Upon activation of JAKs through cytokine stimulation, receptor associated and unphosphorylated STATs are phosphorylated (pYSTAT), which subsequently results in parallel dimerization and translocation to the nucleus to activate gene transcription. STATs regulate gene expression by binding to cognate GAS sites located in STAT-controlled regulatory sites. Common target genes (c-Myc, BCL-2, and D-type cyclins) bind any STAT member in different cell types. STAT-controlled cell-specific-binding sites coincide with cell-specific transcription factors such as C/EBP, HNF4/6, and FOX1/2 in liver tissue and adipocytes as exemplified

The occupation of STAT-binding sites is cell-type specific. In fact, STAT binding is generally enriched in genes that are particularly important for the respective cell type. Bioinformatics analysis of murine and human ChIP-seq data estimated up to ~100,000 sequences occupied by STATs in cells that display high STAT activity (e.g., T cells, macrophages, and hepatocytes), but binding sites were up to 20-fold lower in cell lines with less STAT abundancy (e.g., MEFs and B cells), where ~94% of such sites contained a GAS-like core sequence [19]. Interestingly, bioinformatics studies have also revealed that different transcription factors can bind to the same cis-regulatory elements as STATs [20]. Regulation of specific gene loci via association of STATs with tissue-specific coactivators or corepressors comprises a mechanism by which activation of distinct STAT family members by different cytokines uniquely changes transcription (Fig. 2). Moreover, the ability of STATs to access specific GAS sites could be pre-determined by the cell context via the chromatin status [21]. However, the overall mechanism, its association with PTMs and PPIs, interconnection with other core cancer pathways or connection to metabolism is poorly understood.

The use of various transgenic mice and cell types for extensive analysis of genomic STAT5-binding patterns led to the identification of binding motifs for different transcription factors, which are enriched around the center of STAT5-binding sites (Fig. 2). These include the C/EBP (CCAAT/enhancer-binding protein) family members, which were described to interact cooperatively with STAT5 in adipocytes [22]. In particular, C/EBPα/β/δ were found to highly occupy STAT5-binding sites in mesenchymal or epithelial cells [19]. In hepatocytes, binding motifs of C/EBPα and hepatocyte nuclear factor (HNF) family members, including HNF4/6, significantly coincide with STAT5-binding sites near liver sex-specific genes such as the major urinary protein downstream of GH signaling [19]. Also, binding sites of Forkhead box proteins FOXA1 and FOXA2, which are key regulators in initiating liver specification [23], coincide with C/EBPα and STAT5 binding. FOXA proteins are particularly interesting since they have been described as “pioneer factors” for cell-type-specific transcriptional regulation. As such, they are involved in actively opening the local chromatin to allow other transcription factors to bind. Besides other factors like PBX-1, GREB1, or AP2-δ, FOXA1 has been linked to the modulation of nuclear hormone receptor signaling [24]. Interestingly, STAT5 is also able to interact with the glucocorticoid receptor via its N-terminal oligomerization domain, as well as other nuclear hormone receptors such as the estrogen, progesterone, or androgen receptors. Thus, STAT5 might also act as a pioneer factor similarly to FOXA1, to enhance the binding of key regulators to chromatin in different cell types.

Further interactions of STAT5 with chromatin-binding proteins or other transcription factors such as nuclear factor kappa B (NFκB), the ubiquitously expressed octamer-binding factor 1 (OCT1) and the more B-cell-restricted OCT2 transcription factors were shown. Furthermore, centrosomal P4.1-associated protein (CPAP) was reported to act as a STAT5 coactivator to enhance transcription [25]. These protein–protein interactions antagonize chronic inflammation [26] in intestinal epithelial, are required for cell cycle progression [27], and augment STAT-mediated transcriptional activity [25]. Overall, the cooperative activity of STATs with associated transcription factors appears to control cell-type-specific genes, while the accessibility of their target GAS sites seems to be pre-determined by chromatin configurations. Future studies will be required to elucidate which transcription factors act as pioneer factors that recruit co-transcription factors and/or influence chromatin modifications.

## Antagonistic regulation of STAT5 and BCL6 with consequences for target gene and STAT3/5 locus control

B cell lymphoma protein 6 (BCL6) is an evolutionarily conserved zinc finger transcription factor, which functions as a transcriptional repressor and has essential roles in germinal center B cell differentiation, self-renewal of memory B cells, as well as in the development of follicular helper T (T_FH_) cells [28]. BCL6 is found to be highly expressed in follicular lymphoma and Burkitt’s lymphoma, and its locus is frequently translocated and hypermutated in diffuse large B cell lymphoma (DLBCL) and nodular lymphocyte predominant Hodgkin lymphoma [29]. Intriguingly, STAT5B is also overexpressed in DLBCL [30], and STAT5 is constitutively active in some DLBCL cell lines [31], suggesting that both STAT5 and BCL6 may be important determinants in DLBCL pathogenesis.

**Fig. 3:**
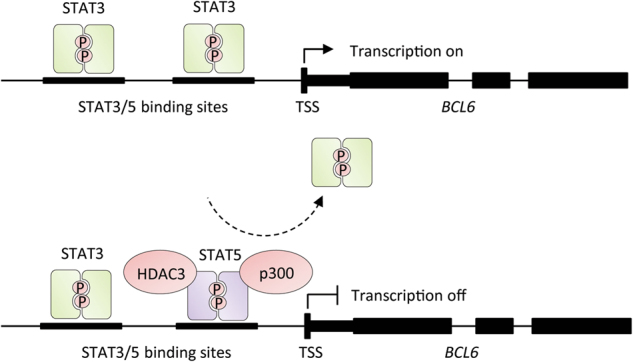
STAT5 and STAT3 compete for binding sites to regulate the expression of the oncogenic transcriptional modulator BCL6. STAT3 increases expression of BCL6 and enhances recruitment of RNA polymerase II phosphorylated at a site associated with transcriptional initiation and elongation. STAT5, in contrast, represses BCL6 expression below basal levels and decreases the association of RNA polymerase II at the gene loci. Furthermore, BCL6 repression mediated by STAT5 is dominant over STAT3-mediated induction. STAT5 exerts this effect by displacing STAT3 from one of the two regulatory regions to which it binds

The roles of STAT5A and STAT5B in the regulation of BCL6 expression are quite controversial. It has been demonstrated that STAT5B upregulates BCL6 in a subset of germinal center cells [32]. On the contrary, STAT5B represses BCL6 in liver epithelial cell lines by interaction with both p300 and HDAC3 [33]. Similarly, STAT5A represses BCL6 expression at the transcriptional level in breast cancer cell lines [34]. STAT3 was found to increase the expression of BCL6 in breast cancer cells; however, the STAT5-mediated repression of BCL6 in these cells was dominant, because STAT5 displaced STAT3 from the shared DNA-binding site [13] (Fig. 3). Repression of BCL6 by increased binding of STAT5 to the BCL6 promoter has also been demonstrated in TH1 cells and natural killer (NK) cells after interleukin-2 (IL-2) stimulation [35]. Again, STAT3 binding was found to be reduced, suggesting competition between STAT5 and STAT3 [35]. Furthermore, it has been reported that STAT5 negatively regulates T_FH_ cell generation due to upregulation of BLIMP-1, which results in repression of BCL6 expression [36]. Since BCL6 and BLIMP-1 reciprocally repress each other, it is possible that STAT5 represses BCL6 expression, which allows BLIMP-1 to be upregulated, thereby preventing T_FH_ generation. A recent analysis of BCL6-binding sites in T_FH_ cells revealed shared BCL6 and STAT5-binding sequences, as well as reduced IL-7 receptor/STAT5 signaling by BCL6 [37]. These reports suggest that STAT5 and STAT3 modulation of BCL6 expression may affect helper T cell lineages based upon BCL6 regulation, and further support a role for STAT5 and STAT3 competing to regulate BCL6 expression.

Recently, it was shown that BCL6 serves as a male-specific transcription factor mediating GH-regulated sexual dimorphism of gene expression in the liver [33]. Specifically, BCL6 binding was preferentially associated with repression of female-biased STAT5 targets in male liver. This suggests that STAT5 and BCL6 have opposing roles in the liver via reciprocal occupancy of the same DNA regulatory sequences [38]. This relationship is most likely a result of GH-activated STAT5B displacing BCL6 on a shared BCL6/STAT5 motif resulting in the expression of sex-specific genes. However, the mechanism of repression or activation by STAT5 is currently unknown. Most likely, the negative regulation by BCL6 and positive regulation by STAT5B involves interactions with epigenetic modifiers such as NCoR1/2, BCoR, p300/CBP, or NCoA-1 [39] as described below.

## Persistent STAT5 activation downstream of oncogenic proteins

Cytokine-dependent STAT3 and STAT5 activation and effects on chromatin are physiologically transient. In contrast, cells that harbor STAT5-activating mutated or modified oncoproteins, like BCR-ABL, JAK2 V617F, mutant MPL (thrombopoietin receptor), and mutant calreticulin in myeloproliferative neoplasms will exhibit persistent activation of STAT5, with high constant levels in the nucleus [40]. Both BCR-ABL positive chronic myeloid leukemia and BCR-ABL negative MPNs are dependent on STAT5 signaling [41]. Chromatin immunoprecipitation (ChIP) and sequencing or ChIP on chip have shown that in cells with persistently activated STAT5 target genes are not identical to those in cytokine-activated cells, with a greater number of low-affinity GAS sites occupied by persistently activated STAT5 [42]. An interesting situation is represented by a group of genes pathologcally overexpressed in MPN cells, which are co-regulated by persistently activated STAT5 in JAK2 V617F cells together with p53 or mutated p53 [43]. Among such targets is LPP (lipoma-preferred partner) that hosts in an intron microRNA-28, which targets the MPL mRNA and inhibits pro-platelet formation [44]. In human erythroleukemia cells, which are homozygous for JAK2 V617F and harbor p53 M133K mutation, downmodulation of p53 or inhibition of STAT5 activation prevents induction of LPP and similarly regulated genes; recruitment of p53 M133K is dependent on STAT5 chromatin binding, while downmodulation of p53 still allows STAT5 chromatin binding. This example illustrates a potential mechanism whereby presence of activated STAT5 on chromatin possibly at low-affinity sites might open up the chromatin for recruitment of different transcription factors or epigenetic regulators.

## Gene transcription and chromatin remodeling by STAT3/5

**Fig. 4:**
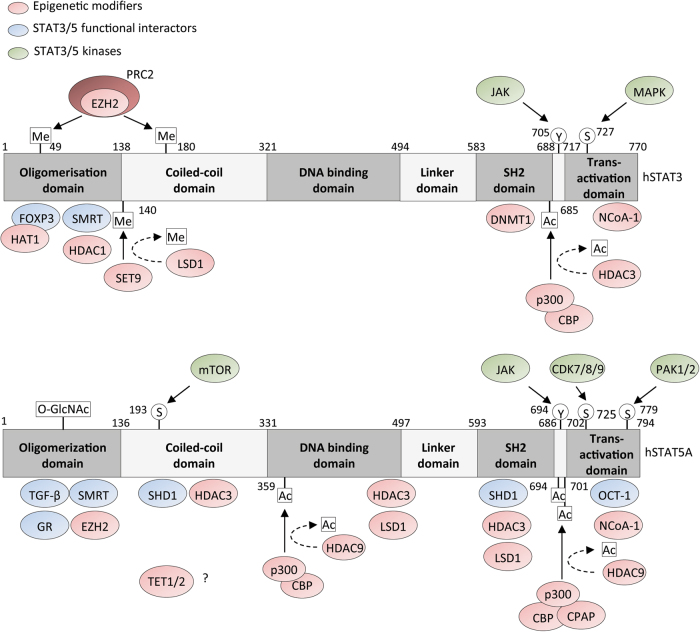
Binding sites of proteins that physically interact with STAT3 and STAT5. Various interacting proteins have been shown to activate or transcriptionally modulate STAT3 and STAT5 (green and blue proteins, respectively) or act as epigenetic modifiers to influence the chromatin landscape in the vicinity of STAT-binding sites

Since the STATs were discovered it has become increasingly evident that, in addition to their binding to GAS elements, epigenetic regulation is a crucial and dynamic part of their gene regulation activity. In response to DNA element binding of transcriptional activators or repressors, the modification of histones by methylation, acetylation, and phosphorylation results in important changes to chromatin structure regulating gene transcription [45]. Histone modifications such as histone H3 lysine 4 acetylation (H3K4ac) and histone H3 lysine 27 acetylation (H3K27ac) favor transcription factor binding and the formation of initiation and elongation complexes. Histone H3 lysine 4 trimethylation (H3K4me3) and H3 lysine 27 trimethylation (H3K27me3) at gene promoters are associated with gene activation and repression, respectively. Moreover, dimethylated H3K4 as well as di- and trimethylated H3K36 have been detected at sites of transcriptionally active chromatin. Many key developmental genes have bivalent modifications where large domains of repressive H3K27me3 mark coexist with small domains of activating H3K4me3 modifications. Importantly, it is now evident that promoter-bound transcription factors such as the STATs are also modified by histone-modifying enzymes, which has important consequences for target gene transcription (Fig. 4).

**Fig. 5:**
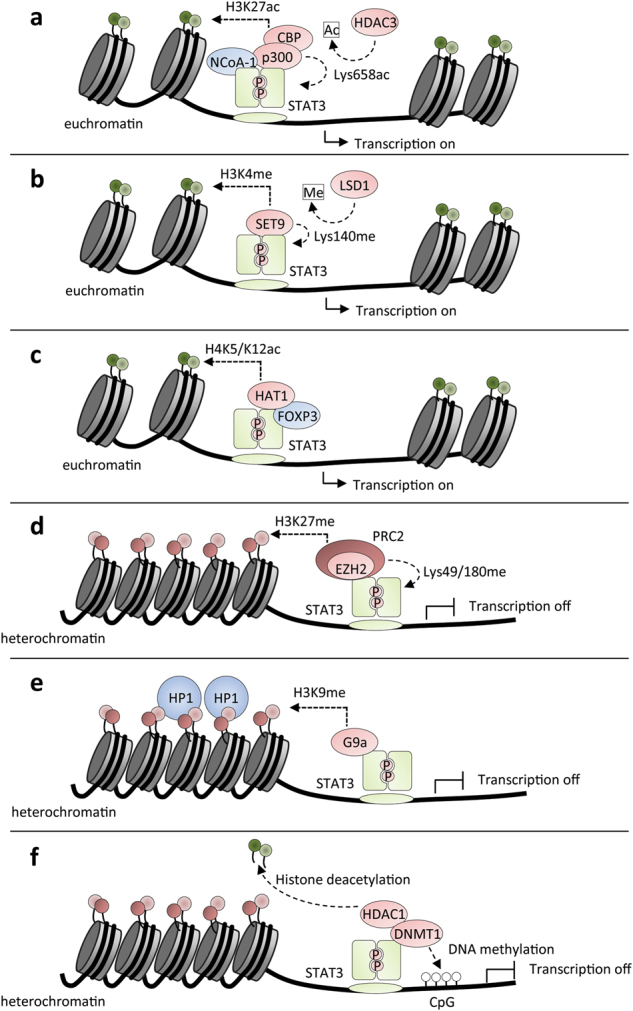
Chromatin remodeling by the transcription factor STAT3. In response to binding of transcriptional activators or repressors to STAT3, modification of STAT3 itself or nearby histones by methylation or acetylation results in important changes of the chromatin structure that regulate gene transcription

STAT3 acetylation on Lys685 within the SH2 domain by the histone acetyltransferase (HAT) p300/CBP promotes STAT3 dimerization, DNA binding, and transcriptional activation in human liver and prostate cancer cell lines [46] (Fig. 5a). However, more recent data have suggested that this modification may play a more important role in unphosphorylated STAT3 (uSTAT3) regulated gene expression [47]. Additional p300/CBP-mediated acetylation sites have also been reported on STAT3 in hepatocytes, where again these modifications seem to promote STAT3 signaling and target gene expression [48]. Deacetylation by HDAC3, and to a lesser extent, HDAC1 and HDAC2, inhibits transcription of STAT3 target genes [49]. Promoter-bound STAT3 can also be methylated at Lys140 in an IL-6-dependent manner by the histone methylase SET9 in human colon cancer cells, and this modification appears to reduce STAT3 binding to DNA, consequently reducing target gene transcription [50] (Fig. 5b). This modification is removed by recruitment of the histone demethylase LSD1 [50]. In regulatory T (T_reg_) cells, FOXP3 acts as a co-transcription factor that facilitates STAT3-mediated IL-10 expression by recruiting HAT1 to the *IL-10* locus [51] (Fig. 5c). Recruitment of HAT1 results in epigenetic modifications to the *IL-10* promoter, creating space for subsequent docking of STAT3–FOXP3 complexes.

Notably, STAT3 can also be methylated on Lys49 and Lys180 by EZH2, the lysine methyltransferase subunit of the polycomb repressive complex 2 (PRC2) in glioblastoma, colon cancer, and breast cancer cell lines [52, 53] (Fig. 5d). Methylation of STAT3 by EZH2 appears to lie downstream of AKT signaling and was shown to increase STAT3 transcriptional activity [52, 53]. Given that STAT3 is an important oncogenic transcription factor, and EZH2 is found to be mutated in many cancers, the interplay between these two proteins raises the possibility that targeting the STAT3-EZH2 axis might be beneficial to arrest or kill cancer cells. STAT3 has also been implicated in regulating epigenetic modifications and chromatin accessibility, adding another level of complexity to its role in transcriptional regulation. STAT3 was found to regulate H3K4 trimethylation, a mark of active transcription, at target gene loci in T cells undergoing Th17 cell differentiation [54]. Furthermore, STAT3 can recruit the histone methyltransferase G9a to form a repressor complex that facilitates H3K9 dimethylation gene silencing marks at the promoter of *miR-200c*, in leptin-treated breast cancer cells [55] (Fig. 5e). Interestingly, it was recently reported that STAT3 can bind to the promoter region of the *EZH2* gene in gastric cancer cells, implicating STAT3 as direct regulator of EZH2 [56]. However, it remains to be determined whether this is a general or cell-type-specific phenomenon, and further work will be required to examine potential STAT3-dependent global changes to histone methylation via regulation of EZH2.

Transcriptional repression is mediated in part by non-DNA-binding corepressors. The corepressors NCoR and silencing mediator for retinoid and thyroid receptors (SMRT) complex were originally identified to associate with nuclear hormone receptors thereby conferring transcriptional repression and subsequently has been shown to be recruited to many classes of transcription factors and is also a component of multiple protein complexes containing HDAC proteins [57]. This association with HDAC activity provides an important mechanism that allows DNA-binding proteins for interaction with NCoR/SMRT to repress transcription of specific target genes. Recruitment of SMRT associated with HDAC by STAT3 leads to the transcriptional inactivation of STAT3 and consequent downregulation of IL-6-mediated multiple myeloma cell growth and gene expression [58].

STAT3 also mediates oncogenesis by recruiting DNA methyltransferase 1 (DNMT1) to gene promoters to silence tumor suppressor genes, such as *PTPN6*, *IL-2Rγ*, *CDKN2A, DLEC1*, and *STAT1* by CpG methylation in malignant T lymphocytes and breast cancer cells [59, 60] (Fig. 5f). Notably, acetylation of Lys685 on STAT3 has been shown to mediate this interaction with DNMT1 [60]. STAT3 was also shown to occupy the *DNMT1* gene promoter in malignant T cells inducing its expression [61]. This suggests a positive feedback mechanism, as inhibition of DNMT1 resulted in a loss of STAT3 activity, and therefore STAT3 may preserve its persistent activation by inducing DNMT1, which in turn acts to silence negative regulators of STAT3, such as the *PTPN6* gene product SHP-1 [61].

**Fig. 6:**
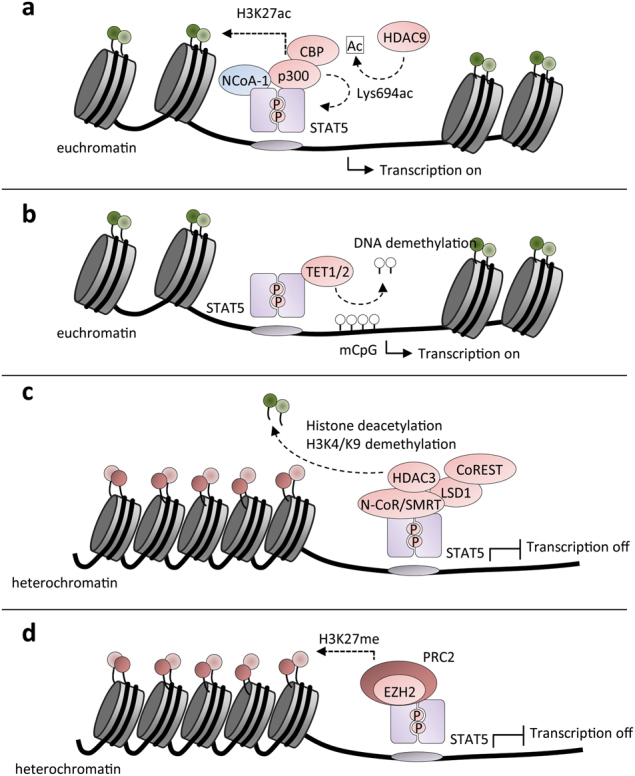
Chromatin remodeling by the transcription factor STAT5. STAT5 regulates the epigenetic landscape by recruitment of activating or repressive proteins conferring epigenetic protein modifications

Like STAT3, STAT5 also regulates the epigenetic landscape in a versatile manner, depending on its interaction partners and transcriptional targets. STAT5-dependent gene activation has been correlated with recruitment of HATs such as NCoA/SRC, or the TUDOR domain coactivators p100 and CBP/p300 to initiate prolactin (PRL)-dependent transcription [62] (Fig. 6a). The PRL-activated receptor signals to STAT5B in mammary epithelial cells, which becomes acetylated at the lysine residue K694 by CBP/p300 and undergoes enhanced dimerization [63]. Furthermore, IL-7 signaling leads to STAT5A acetylation at lysine K696, indicating that acetylation-dependent STAT5 dimerization is also observed in other cytokine signaling pathways [64]. Additionally, STAT5 binding and gene transcription is linked to acetylation of histones H3 and H4 [62, 65]. It was also shown that STAT5 can recruit the DNA demethylases TET1/2 to the *FOXP3* promoter in T_reg_ cells [66] (Fig. 6b). Whether this is a more general epigenetic regulatory mechanism, especially in association with cancer-related constitutive JAK–STAT activity, remains to be shown.

Multiple studies have shown that STAT5 can also function as a transcriptional repressor by recruiting demethylating or deacetylating epigenetic modifiers to specific gene loci [67, 68]. However, this negative transcriptional regulation by STAT5 is thought to be rare. The NCoR1/2 HDAC corepressor complex is probably the most important negative regulator directly interacting with STAT5 [69]. NCoR associates with SHD1, which in turn interacts with STAT5 and represses STAT5-mediated transcription in a cytokine-dependent manner in melanoma [70]. Interestingly, two hotspot mutations in the coiled-coil domain of STAT5A or STAT5B were found in human gastrointestinal cancers [71], suggesting a perturbation of corepressor complex binding to STAT5 [69]. Furthermore, it was reported that recruitment of the corepressor SMRT to STAT5 promoter regions can repress gene expression in response to IL-3 in murine 32D cells [69]. SMRT was found to interact with both STAT5A and STAT5B. Furthermore, due to the fact that the HDAC inhibitor TSA could re-activate target gene transcription, it was concluded that HDACs mediate this process of inhibiting STAT5-dependent transcription [69].

Additionally, both lysine-specific histone demethylase 1A (LSD1) and HDAC3 exert transcriptional regulation of STAT5A targets and facilitate target gene repression by either deacetylation or histone demethylation, respectively (Fig. 6c). Like STAT3, STAT5 is known to interact with SMRT in murine 32D cells [69]. LSD1 forms a complex with corepressor REST (CoREST) [72] and is reported to interact with HDAC3 [73] or NCoR [74]. Considering these various reported PPIs, it is possible that STAT5 exists in complexes containing SMRT/NCoR-HDAC3 and/or LSD1. Furthermore, it was shown that STAT5 is able to recruit EZH2 via direct N-terminal binding in pre-B cells, thereby initiating the formation of H3K27me3 repressive chromatin [68] (Fig. 6d). Although EZH2 was shown to directly methylate STAT3, it has not been found to methylate STAT5 [52].

Intestinal STAT5 has an important function in maintaining genome integrity, which was shown for gamma irradiation-mediated intestinal crypt damage. This damage was more severe upon complete STAT5 loss, and could be antagonized by inducible STAT5 expression [75]. pYSTAT5 could repress BMI1, an essential transcription factor within the polycomb repressor complex 1 (PRC1) that is involved in transcriptional regulation of intestinal epithelial stem and progenitor cells [75]. Notably, STAT5 is also able to directly regulate the expression of epigenetic regulators. It was shown that STAT5 directly binds to the promoter region of the DNA methyltransferase *DNMT3A* in human CD34^+^ AML cells and thereby increases its transcriptional activity, which leads to methylation and thus transcriptional silencing of the tumor suppressor *PTEN* [76]. On the contrary, an association between STAT5 and DNMT3A in T_reg_ cells could not be shown [77].

These results suggest a mechanism that governs the switch between recruitment of coactivators versus recruitment of corepressors by STAT5. Attractive alternatives are either dimers versus tetramers or differential PTMs of STAT5 discriminating between coactivator or corepressor recruitment. Graded pYSTAT5 levels are likely key determinants in opening or closing gene loci. Upon cytokine-dependent activation, STAT5 not only binds to canonical DNA-binding sites as a dimer, but is also able to increase its transcriptional repertoire through binding to tandem repeats of such binding sites as a homo- or heterooligomer with STAT1/3/4 [17]. Oligomer formation stabilizes the binding of STAT dimers to tandem low-affinity sites by decreasing the off-rate of the complex [78]. This can cause an amplification of ongoing transcription [79]. Additionally, tetramers offer a different protein surface accessibility, which allows selective recruitment of other transcription factors or coactivators [80]. It was demonstrated that the interaction of STAT5 with EZH2 is dependent on tetramer formation, while STAT5 dimers lack this repressive function [68]. It can be envisaged that the oligomer composition determines STAT-cofactor interactions at distinct target genes. Detailed mapping analyses of STAT5 coactivators or corepressors, as well as interaction studies in specific cancer types and their consequences for STAT5-regulated biology, remain enigmatic. Epigenetic modifiers such as HATs, EZH2, SMRT, and TET1/2 are frequently mutated in human cancer, specifically in leukemia/lymphoma, carcinomas, and sarcomas, all of which are associated with severely altered transcription. It will be essential to determine how these mutations influence their interaction with STAT dimers or oligomers to shape chromatin and the cancer genome.

## Impact of non-tyrosine phosphorylated STATs on chromatin formation

Prior to activation, uSTAT is maintained as a pre-formed anti-parallel homo- or heterodimer in the cytoplasm, via interaction between the coiled-coil domains [81]. Notably, phosphorylated DNA-bound dimers and uSTAT dimers were shown to be quite distinctive in their structure, indicating different functionalities. Anti-parallel STAT dimers are bound to the cytoplasmic tails of cytokine receptors [82] via the N-terminal domain. STAT5A docking as anti-parallel dimers prevents its autoactivation, whereas deletion of the N-terminal domain renders it persistently active [83], which is not the case for the STAT5B N-domain upon deletion [84].

STAT proteins have three different functions in the non-tyrosine phosphorylated state [85]: (i) as transcription factors, and modifiers of transcription factors in the case of uSTAT1 and uSTAT3 [86], (ii) as mitochondrial effectors such as in the case of serine phosphorylated STAT3 [87], and (iii) as effectors of chromatin topology such as in the case of STAT5A [88]. As reviewed recently, stimulation of STAT1 and STAT3 through interferons (IFNs) and IL-6, respectively, induces increased expression and activity of each protein [86]. While the initial cellular response depending on STAT tyrosine phosphorylation is quite rapidly downregulated through dephosphorylation by SOCS proteins, increased amounts of uSTAT1/3 persist for several days. Notably, it has been shown that pYSTAT1/3 and uSTAT1/3 regulate a distinct set of genes. In breast cancer cells, uSTAT3 regulates genes with well-described roles in cancer, such as muscle RAS (MRAS) and MET, thus potentially contributing to oncogenesis [89]. uSTAT1 prolongs the expression of a subset of IFN-induced genes in combination with uSTAT2 and IRF9 [90]. Additional transcription-related functions of uSTATs include an increased expression of uSTAT3 upon angiotensin II stimulation that leads to angiotensin II-induced cardiac hypertrophy [91], and basal regulation of IFN-activated promoters by constitutive uSTAT2 binding prior to IFN stimulation [92].

**Fig. 7:**
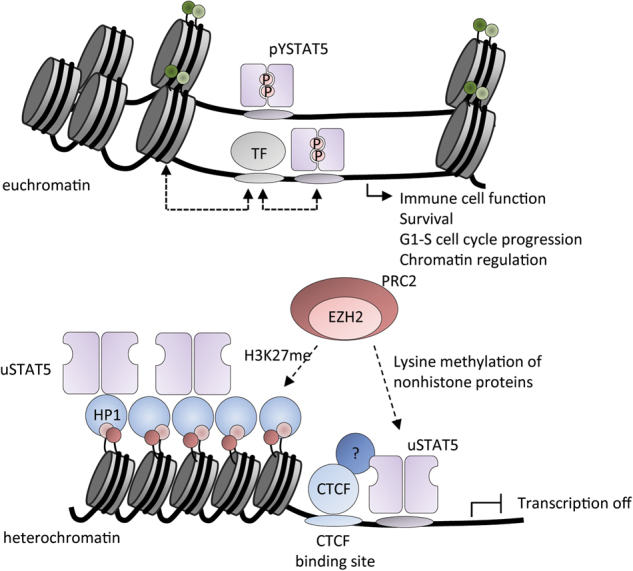
uSTAT5 represses transcription and influences chromatin topology. Activation of canonical STAT5 signaling leads to transcription of common target genes regulating immune cell function, survival, proliferation, and chromatin regulation. uSTAT5, however, is involved in chromatin compaction by interaction with HP-1 and transcriptional silencing by indirect interaction with CTCF

Besides transcriptional regulation, uSTATs might have important roles in cellular compartments other than the nucleus, including mitochondria, Golgi apparatus, and endoplasmic reticulum (ER). Recent work highlights important roles of serine phosphorylated STAT3 in the mitochondria, where it supports RAS-dependent oncogenic transformation in myeloproliferative neoplasms and participates in cellular respiration [93, 94]. Similarly, uSTAT5 was associated with the Golgi apparatus and the ER, as knockdown of STAT5 leads to a dramatic destabilization of these organelles. Additionally, an increasing number of reports demonstrate that T cells employ uSTAT5 for diverse functions in the cytoplasm, mitochondria, and nucleus [95–97]. uSTAT5A was also shown to be involved in heterochromatin compaction through interaction with heterochromatin protein 1α (HP1α) in human cancer cell lines [88] (Fig. 7). The authors proposed that uSTAT5A stabilizes heterochromatin, thereby suppressing tumor cell growth through epigenetic interaction of STAT5A through a PVVVI motif with HP1α bound to H3K9me. Interestingly, STAT5A genes from different species contain an HP1-binding motif, PxVxI, in the DNA-binding domain around amino acid position 467–472, which is conserved to *Drosophila* STAT92E [98]. Although there is evidence for these interactions for uSTAT5A, it is unclear whether this is true for uSTAT5B.

A recent study demonstrated that DNA-associated uSTAT5 and CTCF might influence each other’s transcriptional activity [97] (Fig. 7). CTCF is an architectural protein that binds to topologically associated chromatin domains in the vicinity of insulator sequences, which are hubs for open or closed chromatin structures. Together with cohesin subunits, CTCF has a diverse impact on gene transcription mainly through the establishment of long-range chromatin interactions. Interestingly, uSTAT5 co-localizes with CTCF resulting in repression of transcription. However, upon STAT5 activation by thrombopoietin, pYSTAT5 relocates its binding in the genome to STAT consensus sites, which triggers pYSTAT5-driven gene transcription controlling megakaryocyte proliferation, survival, and differentiation [97]. It remains unclear whether uSTAT5 can also co-localize with cohesin subunits, such as STAG2, which are frequently mutated in cancer [99]. Indeed, STAT5 might also be involved in chromatin looping in a similar manner to cohesin, thereby providing mechanical stabilization of the ring-like structures and facilitating docking of other transcriptional regulators. Since both cohesin and STAT5 are additionally linked to DNA damage repair, a closer association of STAT5 with cohesin subunits might be likely. In line with this, a study examining FLT3-ITD-driven AML described enhanced pYSTAT5 signaling upon haploinsufficient loss of *Smc3*, another subunit of multimeric cohesin [100]. Here, the authors demonstrated altered chromatin structure and increased expression of STAT5 target genes upon *Smc3* mutation, with enhanced STAT5 binding at its response elements due to more relaxed/accessible chromatin.

Enhancer selection by STAT5 is cytokine-dependent, and the stability and function of STAT5 at enhancers and promoters is well characterized [101]. However, the ways in which STAT5 can affect surrounding chromatin upon binding to its target sites is less understood. Overall, STATs, when tyrosine phosphorylated, act as chromatin modifiers by providing a platform for chromatin remodeling enzymes, whereas uSTATs are associated with transcriptional repression and prolonged or alternative transcriptional subsets.

## Concluding remarks

STAT3/5 proteins are the predominant oncogenic transcription factors of the JAK–STAT pathway and regulate gene expression in conjunction with other transcriptional regulators. Therefore, their expression levels and activity are crucial factors, in addition to their interaction with other transcription factors and various epigenetic modifiers such as EZH2, TET1/2, DNMT3A, the corepressor, and histone deacetylase NCoR1/2 and the coactivator histone acetyl transferases p300/CBP. How these interactions are sustained in different cell types and how they change the chromatin landscape dependent on cytokine stimulation remains to be investigated. However, the concept of classic cytokine-mediated JAK–STAT signaling will need to be redefined in the era where we begin to understand chromatin dynamics and gene regulation.

Insights into cancer genome landscapes provide evidence that constitutive activation of STAT proteins and epigenetic gene reprogramming are important hallmarks of human cancer initiation, progression, and metastasis. Future studies will be required to elucidate which transcription factors act as pioneer factors that recruit other transcription factors or cofactors/corepressors to shape chromatin, and how they interplay with chromatin regulators. Detailed 3D chromatin architecture and transcription factor binding analyses combined with chromatin proteomic studies will increase our understanding of how the same key molecules participate in different cellular aspects, ranging from physiological processes like survival, differentiation, and senescence, to transformation and cancer progression. Given the clear importance of the JAK–STAT pathway and their interplay with chromatin remodeling enzymes in the initiation and progression of cancer, targeting of STAT3 and/or STAT5 is of high therapeutic relevance. Furthermore, targeting these pathways in combination with inhibitors against epigenetic modifiers could provide novel treatment avenues.
